# Screening and Functional Transformation Analysis of Genes Related to Skeletal Muscle Development in Supplemental-Fed Oula Sheep

**DOI:** 10.3390/ani15203040

**Published:** 2025-10-20

**Authors:** Yumeng Li, Yanhao Wang, Mingyi Yan, Sen Wu, Meng Liu, Rajwali Khan

**Affiliations:** 1 Academy of Animal Science and Veterinary Medicine, Qinghai University, Xining 810016, China; liyum0520@163.com (Y.L.); wyh20000403a@163.com (Y.W.); ac17278321752@163.com (M.L.); 2 Plateau Livestock Genetic Resources Protection and Innovative Utilization Key Laboratory of Qinghai Province, Xining 810016, China; 3 Key Laboratory of Livestock and Poultry Genetics and Breeding on the Qinghai-Tibet Plateau (Qinghai), Ministry of Agriculture and Rural Affairs, Xining 810016, China; 4 Department of Livestock Management, Breeding and Genetics, The University of Agriculture Peshawar, Peshawar 25130, Pakistan; rajwalikhan@aup.edu.pk

**Keywords:** Oula sheep, Tibetan sheep, longissimus dorsi muscle, growth and development, RNA-seq

## Abstract

**Simple Summary:**

To understand how muscle development changes with growth in Tibetan Oula sheep, we investigated the gene expression profiles in the longissimus dorsi muscle of female Oula sheep during the three-month fetal stage, six-month-old lamb stage, and eighteen-month-old adult stage using transcriptomic sequencing. Comparative analysis across these developmental stages revealed that metabolic processes related to energy and nutrient utilization were progressively enhanced with age. Furthermore, we identified several key genes, such as *NDUFAB1*, and signaling pathways, including the PI3K-AKT pathway, that are potentially critical in regulating muscle development. These findings provide deeper insights into the molecular mechanisms underlying muscle development in Oula sheep, offering valuable insights for future breeding strategies and management practices.

**Abstract:**

To investigate the gene regulatory mechanisms underlying muscle development in Oula sheep at different growth stages, under supplementary feeding, particularly the shift in core regulatory mechanisms governing muscle development from the fetal stage to the postnatal period, we conducted transcriptomic sequencing and comparative analysis of the longissimus dorsi muscle collected during the embryonic, lamb, and adult stages. Differentially expressed genes (DEGs) potentially associated with muscle growth and development were identified across various age phases. Furthermore, Short Time-series Expression Miner (STEM) analysis was employed to decipher the temporal expression patterns of these DEGs. The results indicated that metabolic processes related to carbohydrates, energy, and amino acids were enhanced with increasing age in Oula sheep muscle. Comparative analysis between different growth stages revealed that the functional enrichment of DEGs was directly associated with changes in skeletal muscle development, with significant enrichment in biological pathways such as ECM–receptor interaction, PI3K-AKT signaling pathway, and protein digestion and absorption. Additionally, we observed that *PTPRC*, *IL10*, *NDUFAB1*, *BUB1*, *BUB1B*, *CDK1*, *ITGB3*, and *ITGB2* may play pivotal roles in the regulation of muscle growth and development across different stages in Oula sheep. These findings provide theoretical support for the understanding of the genetic regulatory mechanisms underlying muscle development in Oula sheep.

## 1. Introduction

Oula sheep, a unique breed indigenous to the Qinghai–Tibet Plateau, serve as a significant source of income for local herders and provide a substantial supply of meat, milk, and other livestock products to the local population. This breed holds an important position in the livestock industry [1]. The Oula sheep is also the largest native meat sheep breed on the Qinghai–Tibet Plateau. In Qinghai, adult male Oula sheep weigh an average of approximately 83.6 kg, with the heaviest recorded individual reaching 214.3 kg (at age 4, measured in the 2024 survey). Adult female sheep weigh an average of approximately 62.7 kg. However, the high altitude and cold climate of the Qinghai–Tibet Plateau, characterized primarily by alpine meadows, pose considerable challenges owing to seasonal environmental fluctuations. During the harsh winter months, the scarcity of forage severely affects the growth and development of grazing livestock, thereby constraining the development of local animal husbandry [2]. The most prominent biological characteristic of the Oula sheep lies in its possession of special high-altitude adaptation genes, which enable it not only to survive but also to maintain excellent growth and development capabilities in extremely harsh, high-altitude, and oxygen-deficient environments. As a meat-type sheep, Oula sheep are known for their adaptability to coarse feed, high-quality meat, and minimal gamey flavor, which has contributed to increasing market demand in recent years. Therefore, supplementary feeding during the winter months is essential for the growth and development of Oula sheep. It has been reported that the growth of muscle tissue primarily depends on the proliferation and differentiation of muscle fibers, as well as the subsequent increase in the volume of myofibers [3]. In earlier studies, we used WGCNA analysis to demonstrate that supplemental feeding influences muscle development in Oula sheep through glucose metabolism pathways. However, this represents nutritional regulation of muscle development after birth. The mechanisms by which nutritional supply changes from the fetal to postnatal stages regulate muscle development in Oula sheep remain unclear.

The fetal period is crucial for muscle growth and development, during which muscle progenitor cells undergo a series of proliferation and differentiation processes to form myocytes and multinucleated myotubes, which ultimately mature into myofibers. Furthermore, many flavor-related compounds associated with meat quality begin to accumulate during the fetal period [4]. After birth, the number of skeletal muscle fibers in mammals remains constant; thus, the postnatal growth and development of muscle fibers primarily involve an increase in diameter and changes in fiber type [5]. The regulation of muscle growth is essential for understanding meat characteristics, highlighting the importance of studying muscle development in animals after birth.

With the advancement of high-throughput sequencing technologies, the application of molecular biology in animal genetic breeding research has become increasingly prevalent. Transcriptomics aims to compare gene expression changes in cells or tissues under specific conditions or states, thus facilitating the connection between genetic polymorphisms and phenotypes, and regulating phenotypic traits at the genetic level. Numerous studies have used this technology to explore the genetic mechanisms associated with phenotypic traits, including enhancing disease resistance in pigs [6], investigating the influence of genes on cashmere quality in goats [7], and studying follicular development in goat s [8]. For example, Duckett et al. used high-throughput transcriptomic sequencing to examine the regulatory role of miRNAs associated with intramuscular fat deposition in the longissimus dorsi muscle, discovering many relevant transcripts [9]. Additionally, Zhang et al. employed Illumina 2000 sequencing technology to explore how ovariectomy in female goats affects muscle development, illustrating the significance of this approach in understanding muscle growth mechanisms [10].

The expression of various traits and production performance in animals is primarily determined by genetic material and associated regulatory factors, fundamentally resulting from genetic differences. Investigating the differential gene expression at various growth stages can provide a direct reflection of the impact of supplementary feeding on muscle development in Oula sheep. Therefore, our study employs transcriptomic sequencing technology to explore the genetic regulatory mechanisms underlying muscle growth and development in Oula sheep at key time points: the fetal stage, lamb stage, and growing stage. This research aims to provide a theoretical basis for regulating muscle development from a genetic perspective.

## 2. Materials and Methods

### 2.1. Ethical Approval and Selection of Animals

This trial was conducted with the approval of the Qinghai Academy of Animal Husbandry and Veterinary Science (2025-QHMKY-006).

A total of 15 healthy Oula ewes at 6 months of age (lamb stage, LS group, with an average weight of 30.23 kg) and 15 healthy Oula ewes at 18 months of age (growing stage, GS group, with an average weight of 40.77 kg) were selected, with each group having 5 replicates and 3 ewes per replicate. Under identical management conditions, the ewes were pastured on grass from 9 a.m. to 5 p.m., followed by one instance of supplementary feeding after returning from grazing. The dietary composition and nutritional ingredients shown in Table 1. The ewes had free access to water throughout the experiment, which lasted for 60 days. Following the supplementary feeding period, 5 biological replicates were randomly selected for slaughter at each stage, the ewes were slaughtered, and samples of the longissimus dorsi muscle were collected for subsequent analysis. Additionally, 5 Samples of spontaneously aborted fetuses at 3 months of gestation were collected following a synchronized artificial insemination of the donor ewes, then preserved in liquid nitrogen for further experiments.

### 2.2. Library Construction and Sequencing

RNA was extracted from longissimus dorsi muscle tissue samples of Oula sheep of different ages using the TRIzol reagent. Total RNA was extracted using the Eppscience AIPzol Universal Total RNA Rapid Extraction Kit (i-presci scientific, Beijing, China). The extracted RNA was subjected to quality control using a Thermo Fisher Scientific NanoDrop OneC ultra-micro spectrophotometer (Thermo Fisher Scientific, Shanghai, China). The quality standards were as follows: an A260/A280 ratio of 1.7–1.9 and a concentration of 300–500 ng/μL. Electrophoresis was subsequently performed on a 0.5% agarose gel at 120 V and 100 mA for 30 min. After quality control verification with a Thermo Fisher Scientific NanoDrop OneC ultra-micro spectrophotometer, cDNA was synthesized via reverse transcription using the Eppscience UltraScript First-Strand cDNA Synthesis RT-PCR Kit (i-presci scientific, Beijing, China) (OneStep gDNA Removal) on a Bioer TC-96/G/H(b)B Gene Amplifier. Quantitative real-time polymerase chain reaction (qPCR) analysis was subsequently performed using the 2×Real FST SYBR qPCR Mix (High ROX) kit (i-presci scientific, Beijing, China) on a Thermo Fisher Scientific StepOne™ real-time PCR system (Thermo Fisher Scientific, Suzhou, China). cDNA libraries were constructed via reverse transcription. Once the library construction was completed, the insert size of the libraries was assessed by Agilent 5400 (Agilent Technologies (China) Co., Ltd., Beijing, China), and qPCR was performed to ensure the quality of the libraries. Subsequently, sequencing was conducted using the Illumina NovaSeq 6000 platform (Novogene, Beijing, China). The sheep reference genome version, Ovis_aries_rambouillet. Oar_rambouillet_v1.0.cdna.all.fa.gz. The RNA quality control results are shown in Table 2.

### 2.3. Data Quality Control and Sequence Alignment

To ensure data quality and the reliability of the analyses, we performed quality control on the raw data to obtain clean reads. This process involved the removal of reads containing adapters, reads containing undetermined bases (N), and low-quality reads. The low-quality reads were defined as those with a Qphred score of ≤20, where more than 50% of the bases in the read were of low quality. The clean reads obtained were then aligned to the sheep reference genome using HISAT2 (v2.0.5) software, from which the fragments per kilobase of transcript per million mapped reads (FPKM) for each gene were calculated based on gene length, along with the number of reads mapped to each gene.

The data that support the findings of this study have been deposited into the CNGB Sequence Archive (CNSA) of China National GeneBank DataBase (CNGBdb) with accession number CNP0004540.

### 2.4. Differential Gene Analysis

Differential gene expression(FPKM) between the two groups was analyzed using DESeq2 R package (1.42.0), with thresholds set at |log2FC| > 1 and *p* adjust < 0.05 to identify significantly differentially expressed genes. Furthermore, *p* values were adjusted to control the false discovery rate (FDR). Initially, principal Component Analysis (PCA) was initially used to assess whether each group of samples could serve as a model for the subsequent analysis. Trend analysis of the differentially expressed genes was conducted using STEM (Short Time-series Expression Miner version 1.3.13). Subsequently, Gene Ontology (GO) and Kyoto Encyclopedia of Genes and Genomes (KEGG) enrichment analyses were performed on the significantly differentially expressed modules. The roles of differentially expressed genes between any two time points in muscle development were further assessed through GO annotation and KEGG enrichment analysis, allowing for the identification of differentially expressed genes related to muscle development, along with the associated signaling pathways. Protein–protein interaction (PPI) network analysis of the differentially expressed genes was conducted using the STRING database (https://cn.string-db.org/, accessed on 15 January 2024), and visualizations were generated and refined with Cytoscape_v3.10.0.

### 2.5. qPCR Validation

To further ensure the reliability of the sequencing results, we selected eight differentially expressed genes from various stages for validation using quantitative PCR (qPCR): *PTPRC*, *IL10*, *NDUFAB1*, *BUB1*, *CDK1*, *BUB1B*, *ITGB3*, and *ITGB2*. Primers for each gene were designed using the Primer BLAST online tool available on the NCBI website (https://www.ncbi.nlm.nih.gov) and primers were checked with Primer-BLAST. The specific sequences of the primers for each gene are presented in Table 3. The results were illustrated using GraphPad Prism 9.5.

## 3. Results and Analysis

### 3.1. Analysis of Differentially Expressed Genes in Oula Sheep at Different Ages

**Figure 1:**
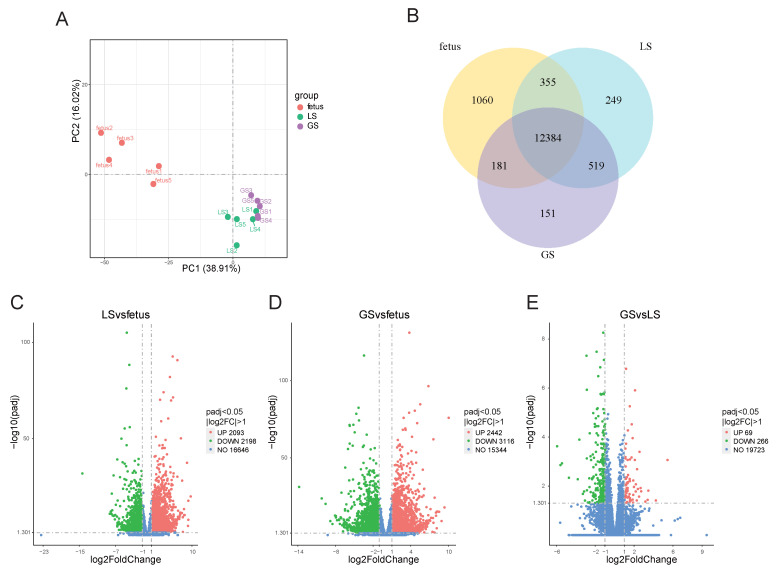
Analysis of gene expression in the longissimus dorsi muscle of Oula sheep. (**A**). PCA of fetus, lamb and growth. (**B**). Venn diagram of three-stage gene expression. (**C**). LS vs. fetus differential gene volcano map. (**D**). GS vs. fetus differential gene volcano map. (**E**). GS vs. LS differential gene volcano map.

Principal component analysis (PCA) was performed on the gene expression levels of Oula sheep at the fetal, lamb, and growing stages, and the resulting PCA plot is presented in Figure 1A (Figure 1A). The plot demonstrates that samples from the three age groups cluster distinctly, indicating that the samples are representative and can serve as a model for further analysis of supplementary feeding in Oula sheep across different age stages. A total of 12,384 genes is expressed across the fetus, LS, and GS. Among these, 1060 genes are specifically expressed in the fetus, 249 genes show specific expression in LS, and 151 genes are uniquely expressed in GS (Figure 1B).

During the transition from fetus to lamb, 2093 genes were significantly upregulated, while 2198 genes were significantly downregulated (Figure 1C). In the transition from fetus to growth, 2442 genes were significantly upregulated, and 3116 genes were significantly downregulated (Figure 1D). Additionally, we analyzed the transition from lamb to growth, where only a small number of differentially expressed genes were identified, with 69 genes significantly upregulated and 266 genes significantly downregulated (Figure 1E).

### 3.2. STEM Analysis of Differential Genes in the Longissimus Dorsi Muscle of Oula Sheep

**Figure 2:**
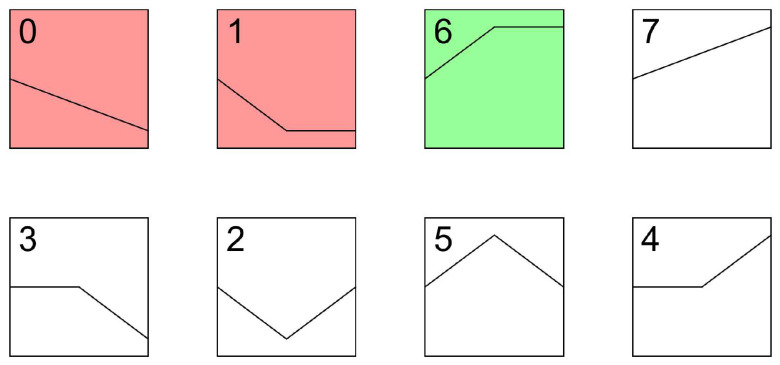
STEM analysis of the longissimus dorsi muscle of Oula sheep. Red represents the decrease in expression levels with age, while green represents the increase in expression levels with age.

Trend analysis of the differentially expressed genes across the fetus, lamb, and growth stages revealed three significantly different modules, as shown in Figure 2. Modules 0 and 1 clustered together, characterized by a general trend of sustained downregulation or initial downregulation followed by stabilization. In contrast, Module 6 consisted of genes that exhibited an initial upregulation followed by stabilization. The remaining modules did not show significant differences in expression patterns.

**Figure 3:**
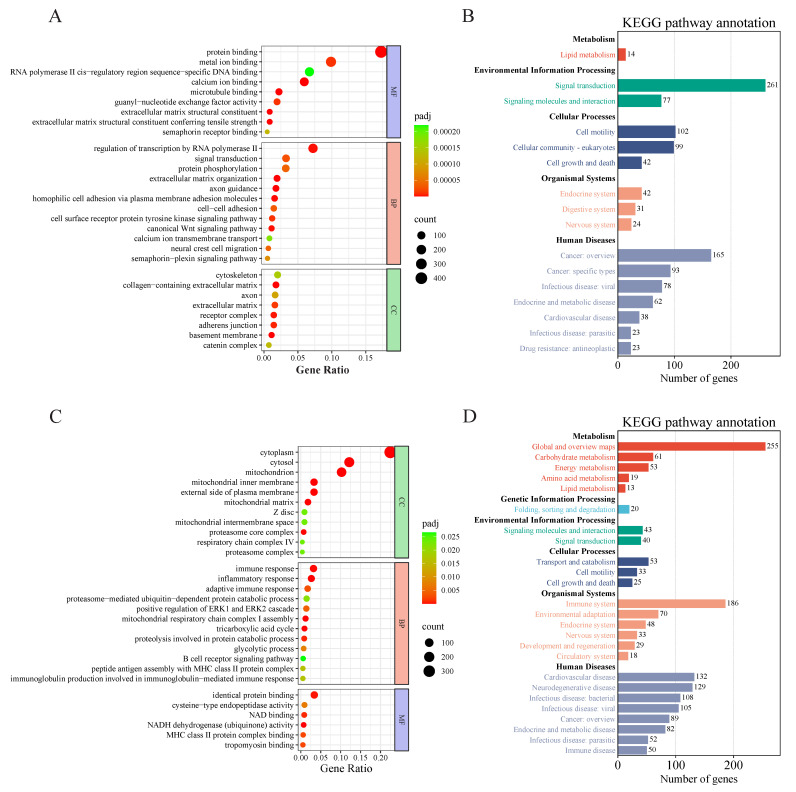
GO and KEGG enrichment analysis of differentially expressed genes in significantly enriched modules of the longissimus dorsi muscle in Oula sheep. (**A**). Dot plot of GO enrichment analysis for differentially expressed genes between Modules 0 and 1. (**B**). Dot chart of KEGG enrichment analysis for differentially expressed genes between Modules 0 and 1. (**C**). Bubble plot of GO enrichment analysis for differentially expressed genes in Module 6. (**D**). Bar chart of KEGG enrichment analysis for differentially expressed genes in Module 6.

The differentially expressed genes from Modules 0 and 1 were combined for GO and KEGG enrichment analyses, and the *p*-values were adjusted for multiple testing using the Benjamini–Hochberg procedure to control the False Discovery Rate (FDR). The GO analysis revealed that these differentially expressed genes were predominantly enriched in processes such as protein binding, metal ion binding, regulation of transcription by RNA polymerase II, protein phosphorylation, and signal transduction (Figure 3A). The KEGG analysis identified significant enrichments in secondary categories including lipid metabolism, signal transduction, signaling molecules and interactions, cell growth and death, and digestive system processes (Figure 3B). Similarly, GO and KEGG enrichment analyses were conducted for the differentially expressed genes in Module 6. The GO results showed that these genes were primarily enriched in cellular components such as the cytoplasm, cytosol, and mitochondrion, as well as in functional processes including protein catabolic processes, the tricarboxylic acid cycle, and glycolysis (Figure 3C). The KEGG analysis indicated that these differential genes were significantly enriched in metabolic pathways related to carbohydrates, energy, amino acids, and lipids, as well as in immune and cellular transport metabolic pathways (Figure 3D).

### 3.3. Differential Gene Enrichment Analysis of LS vs. Fetus

**Figure 4:**
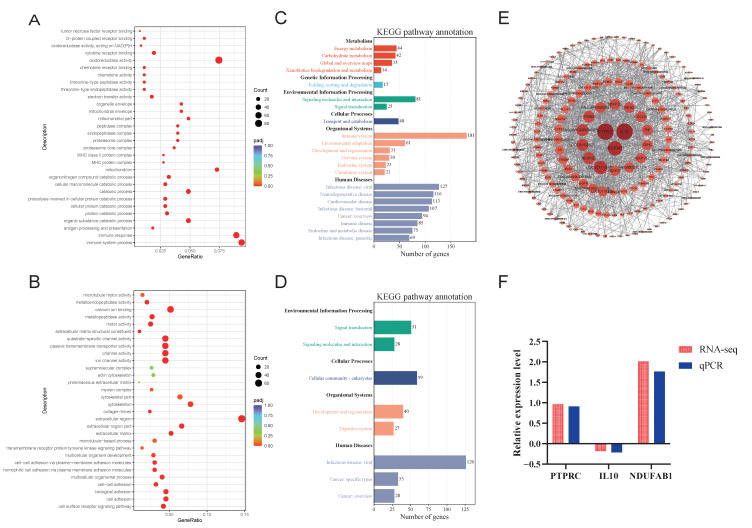
GO and KEGG enrichment analysis of differentially expressed genes in LS vs. fetus. (**A**). Dot plot of GO enrichment analysis for upregulated differentially expressed genes in LS vs. fetus. (**B**). Dot plot of GO enrichment analysis for downregulated differentially expressed genes in LS vs. fetus. (**C**). Bar chart of KEGG enrichment analysis for upregulated differentially expressed genes in LS vs. fetus. (**D**). Bar chart of KEGG enrichment analysis for downregulated differentially expressed genes in LS vs. fetus. (**E**). Protein–protein interaction network of differentially expressed genes in LS vs. fetus. (**F**). qPCR validation of differentially expressed genes in LS vs. fetus.

Next, we conducted analyses of the differentially expressed genes at each developmental stage. For the transition from fetus to lamb, we identified 2093 upregulated and 2198 downregulated genes, and performed GO and KEGG enrichment analyses, with results depicted in Figure 4. In the GO functional annotation, upregulated differentially expressed genes showed significant enrichment in processes such as redox reactions, catabolic processes, and immune responses (Figure 4A). Conversely, downregulated genes were predominantly enriched in extracellular regions and transport and channel activities (Figure 4B). The KEGG enrichment analysis focused on significantly different pathways, revealing that upregulated differential genes were enriched in pathways related to the immune system, energy metabolism (oxidative phosphorylation), carbohydrate metabolism (including the citrate cycle (TCA cycle), fructose and mannose metabolism, glycolysis/gluconeogenesis, propanoate metabolism, pyruvate metabolism, and starch and sucrose metabolism), signaling molecules and interactions, and signal transduction (specifically the NF-kappa B signaling pathway) (Figure 4C). In contrast, downregulated genes were significantly enriched in KEGG pathways associated with the digestive system (protein digestion and absorption), signal transduction (Hedgehog signaling pathway, PI3K-Akt signaling pathway), and cellular community-eukaryotes (signaling pathways regulating pluripotency of stem cells) (Figure 4D).

We also conducted a protein–protein interaction network analysis of the differentially expressed genes, identifying a total of 1521 nodes. From these, due to the large number of gene nodes in the PPI network, so we selected 194 nodes with a degree value greater than 10 for visualization. The results indicated that *PTPRC* was the most central gene, followed by *IL10*, *NDUFAB1*, and others (Figure 4E). Additionally, we validated these differential genes using qPCR, with results demonstrating that the relative expression levels of these three genes were consistent with the trends observed in the transcriptome sequencing results, thereby confirming the reliability of the sequencing data (Figure 4F).

### 3.4. Differential Gene Enrichment Analysis of GS vs. Fetus

**Figure 5:**
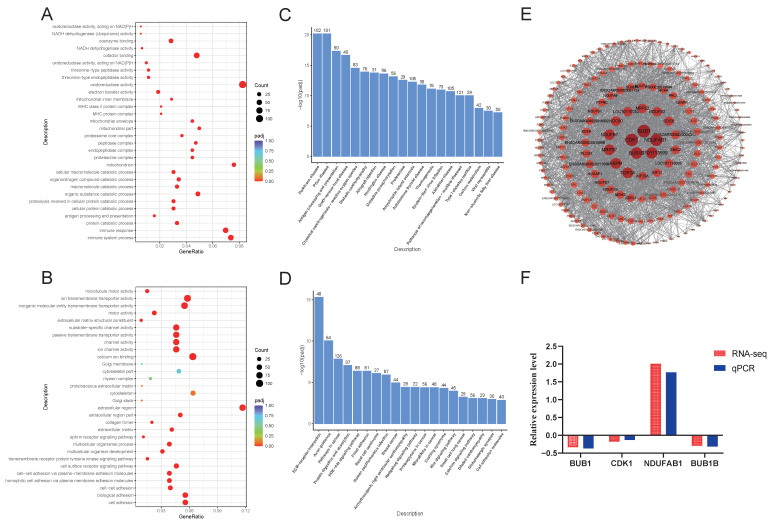
GO and KEGG enrichment analysis of differentially expressed genes in GS vs. fetus. (**A**). Dot plot of GO enrichment analysis for upregulated differentially expressed genes in GS vs. fetus. (**B**). Dot plot of GO enrichment analysis for downregulated differentially expressed genes in GS vs. fetus. (**C**). Bar chart of KEGG enrichment analysis for upregulated differentially expressed genes in GS vs. fetus. (**D**). Bar chart of KEGG enrichment analysis for downregulated differentially expressed genes in GS vs. fetus. (**E**). Protein–protein interaction network of differentially expressed genes in GS vs. fetus. (**F**). qPCR validation of differentially expressed genes in GS vs. fetus.

We also performed GO and KEGG enrichment analyses for the differentially expressed genes in the comparison between GS and the fetus. For the GO analysis, we selected the top 10 most significant terms in the biological process (BP), molecular function (MF), and cellular component (CC) categories for visualization. The results for the upregulated differentially expressed genes showed significant enrichment in immune-related processes, mitochondrion, proteasome complex, endopeptidase complex, peptidase complex, and electron transfer activity (Figure 5A). In contrast, the GO enrichment results for the downregulated genes indicated significant associations with cell adhesion, biological adhesion, calcium ion binding, cell–cell adhesion, ion channel activity, channel activity, and passive transmembrane transporter activity (Figure 5B).

Similarly, KEGG enrichment analysis was conducted for the GS vs. fetus comparison, visualizing the top 20 most significant pathways. The upregulated differentially expressed genes were significantly enriched in oxidative phosphorylation, carbon metabolism, proteasome, antigen processing and presentation, and thermogenesis (Figure 5C). Meanwhile, the downregulated genes showed significant enrichment in pathways associated with the PI3K-Akt signaling pathway, Hedgehog signaling pathway, Wnt signaling pathway, calcium signaling pathway, cell adhesion molecules, focal adhesion, axon guidance, protein digestion and absorption, and glutamatergic synapse (Figure 5D).

We further performed a protein–protein interaction network analysis for the differentially expressed genes in the GS vs. fetus comparison and visualized the results, identifying a total of 2417 interconnected nodes. We selected 184 nodes with a degree value greater than 20 for display. Among these, *BUB1* exhibited the highest degree value of 60, indicating it as the most central gene. Additionally, *CDK1*, *NDUFAB1*, and *BUB1B* had degree values exceeding 55, suggesting their potential roles in the regulation of muscle growth and development in Oula sheep (Figure 5E). Validation of these genes through qPCR demonstrated consistent trends with the RNA-seq results, thus confirming the reliability of the sequencing data (Figure 5F).

### 3.5. Differential Gene Enrichment Analysis of GS vs. LS

**Figure 6:**
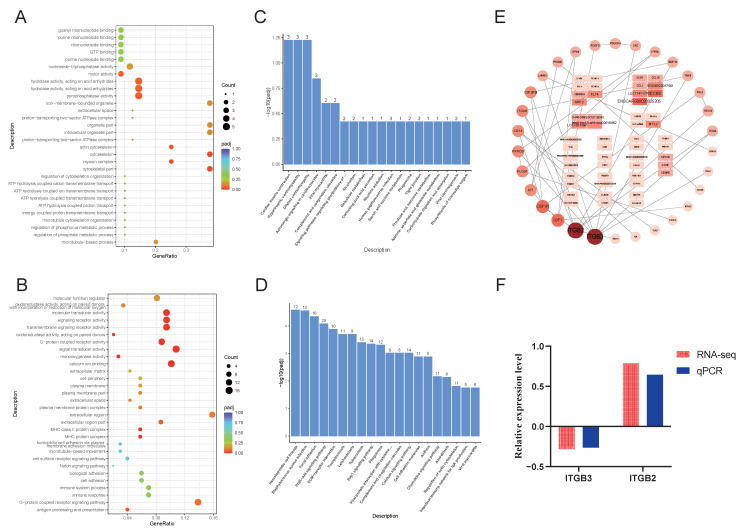
GO and KEGG enrichment analysis of differentially expressed genes in GS vs. LS. (**A**). Dot plot of GO enrichment analysis for upregulated differentially expressed genes in GS vs. LS. (**B**). Dot plot of GO enrichment analysis for downregulated differentially expressed genes in GS vs. LS. (**C**). Bar chart of KEGG enrichment analysis for upregulated differentially expressed genes in GS vs. LS. (**D**). Bar chart of KEGG enrichment analysis for downregulated differentially expressed genes in GS vs. LS. (**E**). Protein–protein interaction network of differentially expressed genes in LS vs. fetus. (**F**). qPCR validation of differentially expressed genes in GS vs. LS.

From the LS to GS stage, the number of significantly differentially expressed genes was lower than that observed in the comparisons of LS vs. fetus and GS vs. fetus. The upregulated differentially expressed genes showed significant enrichment only in the cellular component GO terms related to the cytoskeletal part, myosin complex, cytoskeleton, and actin cytoskeleton (Figure 6A). In contrast, downregulated differentially expressed genes were enriched in GO terms related to the MHC protein complex, calcium ion binding, monooxygenase activity (Figure 6B).

The KEGG enrichment analysis revealed that the upregulated differentially expressed genes were significantly enriched in pathways associated with cardiac muscle contraction, as well as in signaling pathways regulating the pluripotency of stem cells, and galactose metabolism (Figure 6C). Conversely, downregulated differentially expressed genes showed significant enrichment in pathways related to the PI3K-Akt signaling pathway, ECM–receptor interaction, Rap1 signaling pathway, phagosome, and (Figure 6D).

The comparison of lamb to adult stages indicated a significant reduction in the number of differentially expressed genes compared to LS vs. fetus and GS vs. fetus. A protein–protein interaction (PPI) network analysis of these differentially expressed genes revealed that they were mostly loosely connected. Among these, *ITGB3* and *ITGB2* emerged as the genes with the highest degree values, indicating they were relatively more interconnected with other genes (Figure 6E). Furthermore, the expression levels of these genes were consistent with the results obtained from qPCR validation (Figure 6F).

## 4. Discussion

The growth and development of skeletal muscle involve multiple biological processes, including cell proliferation, differentiation, and fusion into muscle fibers. As an important economic trait, the development of animal muscles has garnered significant attention, making the exploration of the molecular mechanisms underlying skeletal muscle cell proliferation and differentiation a major focus of research in the field of biological breeding in recent years [11]. The fetal stage plays a crucial role in muscle development, with the number of muscle fibers being determined as early as the fetal period, as they are the most important component of muscle. After birth, muscle growth primarily results in an increase in the diameter of muscle fibers rather than a change in their quantity [12]. In light of this, the present study conducted RNA-seq analysis on the longissimus dorsi muscle of Oula sheep during the fetal, lamb, and growing stages. Subsequently, differential gene expression analysis was performed on the genes identified at these three stages, followed by STEM trend analysis and GO and KEGG enrichment analysis, to investigate the genes that play key regulatory roles in the longissimus dorsi muscle of Oula sheep at different developmental stages.

In the comparative analysis across different developmental stages, the upregulated DEGs were significantly enriched in pathways related to energy metabolism, carbon metabolism, and immune response. Conversely, the downregulated DEGs were enriched in pathways associated with protein digestion and absorption, ECM–receptor interaction, PI3K-AKT signaling, and Hedgehog signaling. These findings suggest that as Oula sheep mature, their immune system becomes more robust, and energy metabolism, including oxidative phosphorylation, is enhanced to meet developmental demands. Concurrently, the downregulation of pathways like protein digestion and absorption may reflect a developmental shift in nutrient utilization priorities after birth. Our results are consistent with previous studies; for instance, Li et al. [13] also found that differentially expressed genes in the longissimus dorsi of Wannan Hua pigs at various developmental stages were significantly enriched in pathways related to energy metabolism, which aligns with our findings. Similarly, Arora et al. [14] reported that upregulated genes in their study of sheep skeletal muscle were associated with glycogen metabolism and energy metabolism, further supporting our results. The extracellular matrix (ECM), composed of proteins (such as collagen and fibronectin) and polysaccharides (such as glycosaminoglycans, plays a crucial role in tissue growth and development [15]. Research indicates that the ECM is essential for maintaining homeostasis and regulating skeletal muscle development [16]. In our study, the DEGs were also significantly enriched in the ECM–receptor interaction pathway, suggesting that this pathway regulates muscle growth and development in Oula sheep. Moreover, the PI3K-AKT signaling pathway is critical for skeletal muscle development. The phosphoinositide 3-kinase/protein kinase B (PI3K/Akt) signaling pathway, when activated by Akt, is involved in regulating cellular metabolism and promoting the proliferation of myoblasts [17]. In pairwise comparisons of downregulated DEGs across the three stages of Oula sheep development, significant enrichment was observed in the ECM–receptor interaction and PI3K-AKT pathways, indicating that both pathways play regulatory roles throughout the muscle development process. Previous studies have also found that the ECM is involved in skeletal muscle development across various stages [18,19].The growth of animal muscle is dependent on the hypertrophy of muscle fibers, which includes both the growth of myofibrils and the increase in the number of nuclei within muscle fibers [20]. In our research, the downregulated genes enriched in the ECM–receptor interaction and PI3K-AKT signaling pathways suggest that the proliferation of muscle fibers in Oula sheep primarily occurs during the fetal stage, consistent with previous findings. Apart from the pathways associated with abdominal muscles, we also performed a protein–protein interaction analysis of the differential genes, selecting core regulatory genes for muscle growth in Oula sheep based on the degree values of the DEGs.

*PTPRC*, also known as *CD45*, is a gene that encodes a C-type protein tyrosine phosphatase receptor, which is a transmembrane receptor expressed by mature leukocytes. It plays a crucial role in regulating the antigen receptor signaling of T cells and B cells [21,22]. Li et al. [23] found that *PTPRC* not only controls immune function by regulating lymphocyte survival and the activation status of T and B cell antigen receptors but is also linked to lipid metabolism. This suggests that the high expression of the *PTPRC* gene during the lamb stage may not only enhance the immune capacity of Oula sheep postnatally but could also strengthen the absorption and metabolism of nutrients such as lipids. Given that systemic low-grade inflammation is an important mechanism linking obesity and insulin resistance [24], our analysis also focused on key anti-inflammatory mediators. IL10 is an anti-inflammatory cytokine; Gonzalez et al. [25] found that the anti-inflammatory mediator IL-10 correlates with lipid levels, such as triglycerides and free fatty acids, in cells when studying the relationship between inflammation and lipid levels, as well as body weight. Additionally, the research by De Lima-Júnior et al. [26] confirmed that the expression of the *IL10* gene is directly related to mitochondrial function. In our study, IL10 was identified as a core gene in the comparison of the longissimus dorsi muscle between lamb and fetus. Similar to PTPRC, it appears to function at the intersection of immunity and metabolism, potentially influencing energy supply by modulating mitochondrial function. Besides *IL10*, the redox enzyme *NDUFAB1* also has a similar role in mitochondrial function. *NDUFAB1*, also known as mitochondrial acyl carrier protein, acts as an enhancer of mitochondrial metabolism. Zhang et al. [27] directly demonstrated that *NDUFAB1* enhances mitochondrial metabolism and regulates glucose metabolism and insulin signaling, highlighting its significant role in glucose metabolism regulation. The upregulation of *NDUFAB1* expression in the longissimus dorsi muscle of Oula sheep during the lamb and growth stages, compared to the fetal stage, will induce enhanced mitochondrial metabolism, promoting muscle energy metabolism. Previous studies have also identified *NDUFAB1* as a core gene regulating growth and development in the skeletal muscle of chickens [28] and pigs [29]. Furthermore, enhancing mitochondrial function may also provide protective effects on the heart [30].

Meat yield is one of the important traits for evaluating the production capacity of livestock and poultry, and it is also a significant economic indicator for breeding improvement. Genes influencing muscle development can have two regulatory effects: one is positive promotion, and the other is negative suppression [31]. These growth regulatory factors play unique roles in muscle development, modulating cell proliferation and apoptosis in various muscle regions, as well as muscle specificity and sarcomere activation. Our experiments found that the expression of genes such as *BUB1*, *BUB1B*, and *CDK1* in growth was significantly downregulated compared to the fetal stage. Furthermore, PPI analysis revealed that these genes are key regulators of muscle growth and development. BUB1 is a protein kinase involved in the spindle assembly checkpoint, primarily ensuring correct chromosome segregation. Piao et al. [32] discovered that *BUB1* promotes cell proliferation by accelerating the transition from the G2 phase to the M phase of the cell cycle. Zhou et al. [33] found that *BUB1B*, a serine/threonine kinase at the mitotic checkpoint, also influences cell proliferation and migration, significantly correlating with the activation of the cell cycle. Further exploration revealed that *BUB1B* can regulate glycolysis, acting as an activator in glycogen metabolism. Moreover, *BUB1* can inhibit inflammation-induced osteoclastogenesis, thereby playing a crucial role in alleviating bone loss [34]. In this study, the significant downregulation of *BUB1* and *BUB1B* from the fetus to growth stage suggests a notable decline in cell proliferation in adult Oula sheep. The decreased of *BUB1* expression may also indicate potential issues such as impaired bone maintenance in mature individuals. The phosphorylation of cyclin-dependent kinase 1 (*CDK1*) can lead to the phosphorylation of *BUB1*. When *CDK1* is active, its phosphorylation can generate important centromere receptors like *BUB1* among many proteins early in cell proliferation [35,36]. Studies have shown that *CDK1* regulates the proliferation of myogenic cells, muscle fiber hypertrophy, and muscle regeneration, with downregulation of *CDK1* indicating differentiation of myogenic cells. The absence of this gene can impair muscle regeneration [37]. Multiple previous studies have emphasized the regulatory role of *CDK1* in the proliferation of skeletal muscle myogenic cells [38,39,40], which is consistent with our findings. Additionally, in the analysis comparing growth and lamb, *ITGB3* and *ITGB2* were also identified as core genes for muscle development. *ITGB3* is a highly expressed adhesion receptor in osteoclasts that promotes osteogenic differentiation [41,42]. Research has shown that *ITGB3* plays a crucial role in the development of intramuscular fat (marbling) in the longissimus dorsi muscle of cattle [43]. Knockout of *ITGB3* impairs the expression of myogenic genes and disrupts actin organization, leading to compromised myotube formation [44]. In addition to *ITGB3*, *ITGB2* was also identified as a key gene in our study. Li et al. [23] found that *ITGB2* is closely associated with immune cells and has connections to lipid metabolism. *ITGB2* can promote mitochondrial glycolysis and cellular energy supply via the PI3K-AKT-mTOR pathway [45].

In summary, our study elucidates the major signaling pathways and core genes associated with developmental differences in the longissimus dorsi muscle of Oula sheep at various stages. Under supplementary feeding conditions, the *NDUFAB1* gene was identified as a key differentially expressed gene in both the LS vs. fetus and GS vs. fetus comparisons, underscoring its significant role in muscle development. These findings will enhance our understanding of the genetic regulation underlying skeletal muscle growth. However, this study has certain limitations. The transcriptomic analysis was conducted under a specific feeding regime, and the candidate genes and pathways identified require further functional validation through in vitro or in vivo experiments. Future research should employ techniques such as gene knockout or overexpression to definitively confirm the roles of these core genes, including NDUFAB1. Additionally, exploring the synergistic effects of multiple genes and integrating multi-omics data will be crucial for comprehensively understanding the complex regulatory network governing sheep muscle development.

## 5. Conclusions

This study investigated the genetic alterations and potential regulatory mechanisms involved in muscle growth and development of Oula sheep at different ages. The results demonstrated that metabolic processes related to carbohydrates, amino acids, and lipids were enhanced with advancing age. In pairwise comparisons between different age groups, the PI3K-AKT signaling pathway was consistently enriched among the downregulated genes. Genes such as *PTPRC*, *IL10*, and *NDUFAB1* identified in the lamb stage, and *BUB1*, *CDK1*, *NDUFAB1*, and *BUB1B* detected in the growing period, may serve as potential key regulators of muscle growth in Oula sheep. Notably, *NDUFAB1* was identified in both comparisons, suggesting its crucial role in muscle development. This study reveals the cross-stage central role of genes such as *NDUFAB1* and the PI3K-AKT signaling pathway in the muscle development of plateau sheep, providing a novel regulatory perspective for understanding metabolic reprogramming in ruminant muscle. It offers critical targets for the molecular breeding of Oula sheep, whereby early selection or nutritional interventions targeting marker genes like *NDUFAB1* could directly enhance breeding efficiency and meat quality.

## Figures and Tables

**Table 1:** Dietary composition and nutrient levels (dry matter basis).

Ingredients	Contents %	Nutrient Levels ^(2)^	Contents %
Corn	39.92	CP	15.09
Soybean meal	12.99	ME (MJ/kg)	2.62
Wheat bran	7.73	Ca	0.79
Rapeseed cake	6.93	TP	0.4
Limestone	1.42	Lys	0.67
Premix ^(1)^	1.01	Met	0.26
Dried corn stalk	30.00	NDF	33.34
Total	100.00	ADF	19.79

^(1)^ The premix provided the following per kg of diets: Cu 18 mg, Fe 50 mg, Mn 15 mg, Zn 16 mg, I 0.36 mg, Se 0.56 mg, Co 0.08 mg, VA 2000 IU, VD 342 IU, VE 200 IU. ^(2)^ Nutrient levels were calculated values.

**Table 2:** RNA-seq quality control.

Sample	Raw Reads Number	Cleanreads Number	Cleanreadsrate (%)	Clean Q30 Basesrate (%)	GC Content (%)	Mappingratio (%)	Uniquemappingratio (%)
fetus1	47,639,868	45,509,926	95.53	93.84	52.58	96.06	87.25
fetus2	47,164,860	44,679,420	94.73	92.00	52.62	94.36	87.82
fetus3	47,697,680	45,048,138	94.45	92.34	52.31	94.73	87.94
fetus4	45,614,790	42,633,336	93.46	91.94	51.86	94.38	87.19
fetus5	43,742,352	40,576,428	92.76	92.94	51.73	94.94	85.76
LS1	41,212,104	38,519,372	91.71	91.99	51.8	94.64	83.34
LS2	46,013,906	43,545,542	93.24	92.17	49.36	95.43	81.43
LS3	45,748,468	43,155,138	98.15	92.39	52.42	94.87	84.31
LS4	45,241,120	41,792,798	98.15	92.5	49.01	94.89	80.92
LS5	46,274,008	43,830,938	97.26	92.06	51.92	94.95	83.81
GS1	43,475,578	39,881,436	91.73	93.4	51.23	95.01	82.66
GS2	48,228,642	45,179,514	93.68	91.42	50.91	94.66	81.91
GS3	47,582,042	45,289,166	95.18	92.23	51.92	95.32	84.98
GS4	48,922,814	45,936,318	93.90	92.41	50.03	94.93	80.61
GS5	50,433,518	49,632,898	98.41	93.39	52.25	96.45	85.27

**Table 3:** Primers of some differentially expressed genes.

Gene	Accession No.	Primer Sequence (5′-3′)	Tm/°C	Size/bp
*PTPRC*	XM_060396233.1	F CTGCAGAACCCAAAGAATTGGTC R GGATGAGAAGAGGCACATTCCTG	61	111
*IL10*	XM_060395938.1	F GGGAAGGAGACCTCCAGGAT R AGGGGAGAGGCACAGTAGAG	60	122
*NDUFAB1*	XM_027961453.2	F CAGTGCATCCTGGGTCGG R GAAGAGGGTAGTGCTGAGCG	60	136
*BUB1*	XM_012173898.5	F TCAGAGGGTCTTCCCCATTAC R GATCACAGTAAGCTGTCTATCCTG	59	284
*CDK1*	NM_001142508.1	F CCTGCCAAACGAATTTCTGGC R CTGCTCTTGACACAACACAGG	60	117
*BUB1B*	XM_042252264.1	F GGAACAAGGAAGGTCTAAGGGT R ACATGAGTGGTTATGACTTTCTGT	60	299
*ITGB3*	XM_060395754.1	F CATGTATGCGCCATCCTCCT R CGTTCAGCAAGCCTCATCCT	60	128
*ITGB2*	NM_001009485.1	F GAGAAGCTGAGGAACCCCTG R GCTTCCCGACTTCTGTCTCG	60	118
*GAPDH*	XM_015097299.4	F ACGACCACCCTATCCAGGTT R AGGTCCTGGATGGAGGCTT	60	271

## Data Availability

Within this published article, all research data generated or analyzed in the current study are contained.
